# The Signaling Pathways Involved in the Anticonvulsive Effects of the Adenosine A_1_ Receptor

**DOI:** 10.3390/ijms22010320

**Published:** 2020-12-30

**Authors:** Jeroen Spanoghe, Lars E. Larsen, Erine Craey, Simona Manzella, Annelies Van Dycke, Paul Boon, Robrecht Raedt

**Affiliations:** 1 4Brain, Department of Head and Skin, Ghent University, 9000 Ghent, Belgium; Jeroen.Spanoghe@UGent.be (J.S.); LarsEmil.Larsen@UGent.be (L.E.L.); Erine.Craey@UGent.be (E.C.); Simona.Manzella@UGent.be (S.M.); Paul.Boon@UZGent.be (P.B.); 2 Department of Neurology, General Hospital Sint-Jan Bruges, 8000 Bruges, Belgium; Annelies.VanDycke@azsintjan.be

**Keywords:** adenosine, adenosine A_1_ receptor, epilepsy, signaling pathways, neuromodulation, inhibition

## Abstract

Adenosine acts as an endogenous anticonvulsant and seizure terminator in the brain. Many of its anticonvulsive effects are mediated through the activation of the adenosine A_1_ receptor, a G protein-coupled receptor with a wide array of targets. Activating A_1_ receptors is an effective approach to suppress seizures. This review gives an overview of the neuronal targets of the adenosine A_1_ receptor focusing in particular on signaling pathways resulting in neuronal inhibition. These include direct interactions of G protein subunits, the adenyl cyclase pathway and the phospholipase C pathway, which all mediate neuronal hyperpolarization and suppression of synaptic transmission. Additionally, the contribution of the guanyl cyclase and mitogen-activated protein kinase cascades to the seizure-suppressing effects of A_1_ receptor activation are discussed. This review ends with the cautionary note that chronic activation of the A_1_ receptor might have detrimental effects, which will need to be avoided when pursuing A_1_ receptor-based epilepsy therapies.

## 1. Introduction

Epilepsy is a chronic brain disease ranking among the most common neurological disorders with an estimated prevalence of around 1% worldwide [1,2]. First-line treatment consists of pharmacotherapy with anti-epileptic drugs. Despite the development and approval of more than 20 new drugs over the past few decades, about one third of all epilepsy patients cannot be effectively treated this way [3,4]. This significant proportion of patients suffering from drug-resistant epilepsy has been an important drive for the search for new and better epilepsy treatments. In this regard, a lot of research has focused on the role of adenosine in epilepsy, owing to its ability to act as an endogenous seizure terminator and its potent anticonvulsive effects [5,6,7]. A great deal of studies have examined the mechanisms behind the anti-epileptic effects of adenosine and demonstrated that adenosine or adenosine analogues are effective in suppressing epileptic seizures, and this mainly through activation of adenosine A_1_ receptors. Several excellent reviews have been published in recent years describing the current knowledge on the role of adenosine in epilepsy and its therapeutic potential (see references [8,9,10]). The aim of this review is to specifically focus on the inhibitory A_1_ receptors and their downstream signaling pathways, giving an overview of the consequential neuronal effects and how these effects contribute to the seizure suppressing role of adenosine.

## 2. Adenosine in the Central Nervous System

Adenosine is a purine ribonucleoside fulfilling an important role in many physiological processes [11]. It has a general homeostatic role as modulator of cellular metabolism, but in the central nervous system (CNS) it also distinctively functions as a neuromodulator [12]. Adenosine is involved in various neural processes, including the regulation of sleep, arousal, nociception and respiration [13,14,15,16].

**Figure 1:**
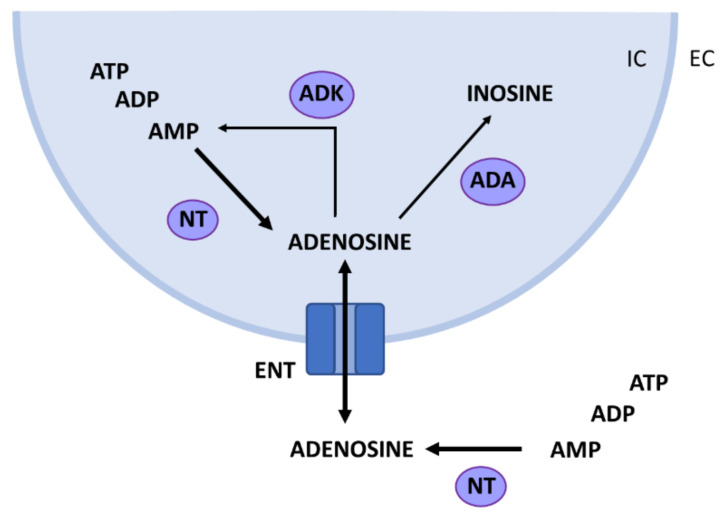
Adenosine metabolism in the brain: intra-(IC) and extracellular (EC) catabolization of adenine nucleotides (ATP, ADP, AMP) by nucleotidases (NT) leads to formation of adenosine. Intracellularly, adenosine deaminase (ADA) breaks down adenosine to inosine and adenosine kinase (ADK) phosphorylates adenosine to AMP. Bidirectional transport of adenosine via equilibrative nucleoside transporters (ENT) equalizes the IC and EC adenosine concentrations.

Adenosine is constitutively present at low concentrations in the brain, with basal extracellular adenosine levels kept in the range of 50–200 nM through enzymatic control [17]. The main source of adenosine in the brain is the intra- and extracellular breakdown of adenine nucleotides by 5′-nucleotidases (Figure 1). Adenine nucleotides released in the extracellular space, such as adenosine triphosphate (ATP) or adenosine monophosphate (AMP), are rapidly converted to adenosine [18]. Intracellularly, the formation of adenosine is linked to the energy consumption of the cell. An increase in cellular workload and in degradation of cytoplasmic ATP leads to increased formation of adenosine, with small intracellular changes in the concentration of ATP resulting in substantial changes in adenosine concentrations relative to its basal levels [12,19]. Adenosine formed intracellularly then exits the cell via equilibrative nucleoside transporters (ENTs), which allow for bidirectional passive transport of adenosine according to the concentration gradient. This way, extracellular adenosine concentration is mainly regulated via two intracellular enzymes: adenosine deaminase (ADA), which catabolizes adenosine to inosine, and adenosine kinase (ADK), which phosphorylates it to AMP [17,20]. Under physiological conditions, ADK is the main regulator of adenosine concentrations, but when concentrations increase in case of energy imbalance ADA exerts a more important role [21].

Extracellular adenosine exerts its modulatory effects via binding to G protein-coupled receptors (GPCRs), of which four subtypes have been characterized: A_1_, A_2A_, A_2B_ and A_3_. These subtypes possess different affinities for adenosine and couple to specific G proteins. The adenosine A_1_ receptor (A_1_R) couples to G_i_ and G_o_ proteins, the adenosine A_2A_ receptor (A_2A_R) couples to G_s_ and G_olf_ proteins, the adenosine A_2B_ receptor (A_2B_R) couples to G_s_ and G_q_ proteins and the adenosine A_3_ receptor (A_3_R) couples to G_i_ and G_q_ proteins [20]. The A_1_ and A_2A_ subtypes are high affinity receptors, with the A_1_R possessing the highest affinity for adenosine. They are the most abundantly expressed adenosine receptors in the CNS, while the A_2B_R and A_3_R have much lower affinities and are only expressed there in comparatively small numbers [13]. Highest CNS expression levels of the A_1_R are found in the neocortex, hippocampus, thalamus, cerebellum and spinal cord. The A_2A_Rs on the other hand are predominantly expressed in the striatum [22].

## 3. Role of Adenosine in Epilepsy

Epilepsy is characterized by the generation of recurrent, unprovoked seizures [23]. These epileptic seizures are disruptions of neurological function caused by excessive or hypersynchronous neuronal activity and can be seen as the consequence of an imbalance between excitation and inhibition in the brain [24]. Excessive excitation and/or deficient inhibition leads to uncontrolled firing of neurons, which causes great metabolic stress during seizures. Consequently, adenosine levels rise and, because of its homeostatic role, adenosine responds as an endogenous anticonvulsant to counter this neuronal hyperactivity.

This link between adenosine and epilepsy was initially discovered 40 years ago. Studies focused on the involvement of adenosine in the regulation of cerebral blood flow showed an increase in adenosine levels during bicuculline-induced seizures [25,26]. Around the same time, the anticonvulsive properties of adenosine were demonstrated for the first time in vitro, in a hippocampal slice model [5], which triggered a wave of studies that confirmed the anticonvulsive effects of adenosine or adenosine analogues both in vitro [27] and in vivo [6,28,29]. The increase in adenosine concentrations during seizures was later also demonstrated in human patients with epilepsy using microdialysis [7]. Here, adenosine was found to reach levels high enough to suppress epileptiform activity in vitro and its concentrations remained elevated for the entire postictal period. These findings further supported the hypothesis that adenosine acts as an endogenous anticonvulsant and seizure terminator.

Soon after the first demonstration of the anticonvulsive properties of adenosine, the importance of A_1_Rs in mediating these effects was suggested [28]. In vitro electrophysiological studies showed that A_1_Rs were responsible for the inhibitory neuromodulatory effects of adenosine [30,31]. In various in vivo studies A_1_R agonists suppressed electrically or chemically induced seizures while A_1_R antagonists conversely aggravated seizure activity [32,33,34,35,36]. Furthermore, the potency of adenosine analogues as anticonvulsants was found to be positively correlated with their affinity for the A_1_R [37]. Besides studies demonstrating the anticonvulsant effects of exogenous activation of A_1_R, experiments with transgenic A_1_R-knock out animals delivered proof of the importance of endogenous adenosine acting on A_1_Rs. Deletion of A_1_Rs in mice leads to spontaneous seizures, increased spreading of induced seizures, aggravated seizure-induced brain damage and even the development of lethal status epilepticus [38,39,40].

Changes in the expression levels of A_1_Rs after seizures also further underline their relevance in relation to seizures and epilepsy. In acute seizure models, there is a clear upregulation of A_1_R expression in a matter of hours after the induction of seizures [41,42,43]. This shows that in an initial response to seizures, there is a potentiation of the anticonvulsive effects of the adenosinergic system by increasing the amount of A_1_Rs. However, the long-term modifications in chronic epilepsy are less straightforward. Multiple studies present contradictory results regarding changes in A_1_R expression in chronic epilepsy (Table 1). In temporal cortex biopsies from temporal lobe epilepsy (TLE) patients, both increases and decreases in A_1_R density have been reported [44,45]. In the latter case, however, biopsies of epileptic cortex were compared to control tissue of post-mortem human brains [45]. Initial studies in chronic seizure models indicated that brain structures affected by seizures displayed a decreased expression of A_1_Rs [46,47,48]. However, later studies also found increases in A_1_Rs and A_1_R mRNA in the epileptic brains of kindled animals [49,50]. The exact reason for these conflicting results remains unclear, they could be in part due to methodological differences between the studies. Nevertheless, it is evident that changes in the expression of A_1_Rs must play a role in epilepsy. An increase in A_1_Rs in case of chronic seizures could, similarly to the response to acute seizures, indicate the presence of an endogenous adaptive mechanism to limit the hyperexcitability of epileptic networks. On the other hand, reports of the loss of A_1_Rs after repeated seizures have led to the hypothesis that, together with maladaptive changes in the metabolism of adenosine (e.g., the overexpression of astroglial ADK [51]), this impairment of the inhibitory modulatory adenosine system is an important aspect in the development and progression of chronic epilepsy. Despite a possible decrease in A_1_R expression levels, administration of A_1_R agonists in chronic epilepsy models is still able to produce robust anticonvulsive effects [36,52]. It is thus very clear that inhibitory A_1_Rs are largely responsible for the contribution of adenosine in epilepsy. For this reason, the following sections of this review will focus on this adenosine receptor subtype.

## 4. A_1_R Structure, Activation and Expression

**Figure 2:**
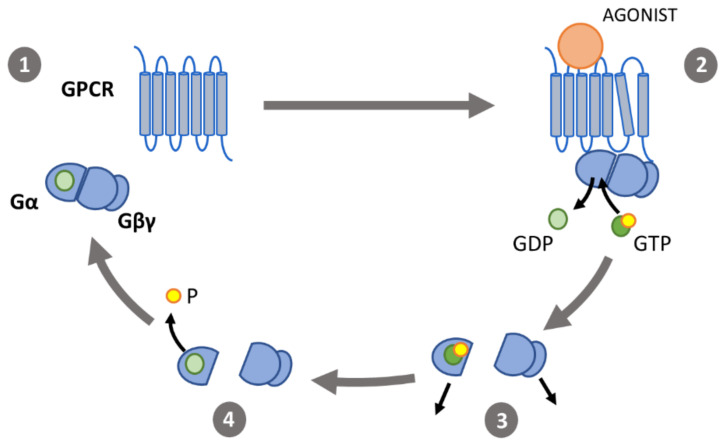
The G protein activation cycle: (**1**) in its inactive state, the α-subunit (Gα) binds guanosine diphosphate (GDP) and forms a heterotrimeric G protein complex with the β- and γ-subunits (Gβγ). (**2**) Binding of an agonist to a G-protein coupled receptor (GPCR) induces conformational changes. The outward movement of transmembrane helix 6 enables interaction of the GPCR with the heterotrimeric G proteins, catalyzing the exchange of GDP for GTP. (**3**) Gα and Gβγ then dissociate and interact with effectors. (**4**) Gα-induced hydrolyzation of GTP to GDP causes the G protein subunits to associate and return to their inactive state.

The A_1_R, together with the other adenosine receptors, belongs to the GPCR superfamily and is further classified into the α subfamily of the rhodopsins (formerly called “class A” of the GPCR superfamily) [53]. It is a glycoprotein with a molecular mass of ~36 kDa and, like all GPCRs, consists of 7 transmembrane α-helices, 3 extracellular and 3 intracellular loops, an extracellular N-terminus and an intracellular C-terminus [53,54]. The first four transmembrane domains of the A_1_R, (from the N-terminus to the end of the second extracellular loop) have been shown to be important for ligand binding and conferring specificity for A_1_-selective agonists/antagonists [55]. More recently, the determination of the crystal structure of the A_1_R in its inactive state has confirmed that conformational differences in these regions, especially the distinct conformation of the second extracellular loop, could underlie the selectivity of ligands for the A_1_ subtype [56]. Binding of an agonist to the A_1_R induces structural changes leading to receptor activation. The overall activation process is similar for all GPCRs and involves the relative rearrangement of transmembrane helices. A key transition during activation is the outward movement of the intracellular part of the transmembrane helix 6 (Figure 2), which has been observed in multiple GPCRs including the adenosine-bound A_1_R [57]. This opens up the cytosolic side of the receptor and enables interaction with G proteins, resulting in a ternary complex between agonist, receptor and G protein. Experiments with fusion proteins of the A_1_R and G protein subunits have indicated that receptor activation is the rate-limiting step in this ternary complex formation, rather than the interaction between the activated receptor and the G protein [58]. The kinetics of this activation process have been studied by looking at conformational changes with fluorescence resonance energy transfer (FRET) sensors. In these studies, receptor activation times were indirectly measured in various GPCRs and were found to be in the range of 30–50 ms [59].

The gene coding for the human A_1_R is located on the long arm of chromosome 1 and contains two separate promotors; A and B [60,61]. This results in two distinct transcripts of the A_1_R gene: transcript α produced by promoter A and transcript β produced by promoter B. Transcript β is found in all tissues expressing A_1_Rs while transcript α is only seen in tissues with high levels of A_1_R expression, such as the brain, testis and kidney [61]. This is due to multiple AUG codons in the 5′-untranslated region of transcript β which hinder protein expression at the post-transcriptional level [62]. In the CNS, A_1_Rs are most abundant in neurons, but A_1_Rs are also expressed by astrocytes, microglia and oligodendrocytes [63]. Receptor distribution varies per region, with the highest densities of A_1_Rs being found in the hippocampus [64]. The subcellular localization has been investigated in rat hippocampal neurons, where A_1_Rs are present extrasynaptically on the membrane of cell bodies, axons and dendrites and synaptically in the active zone of presynaptic terminals and at the postsynaptic density [65,66,67,68].

## 5. A_1_R Signaling

### 5.1. Coupling to G Proteins

G proteins are heterotrimeric complexes composed of a nucleotide-binding α-subunit (Gα) and a dimer containing the β- and γ-subunits (Gβγ). In its inactive state Gα is bound to guanosine diphosphate (GDP) and forms a stable complex with Gβγ. Activated GPCRs can interact with inactive G proteins and catalyze the release of GDP from the Gα subunit, which is rapidly replaced by guanosine triphosphate (GTP). This induces the dissociation of the heterotrimeric complex from the GPCR and a separation of the subunits. The GTP-bound Gα subunit, now in its active state, and the Gβγ subunit are then both free to interact with downstream effectors. Gα, which possesses intrinsic GTPase activity, hydrolyzes the bound GTP to GDP, terminating the signal. Then, Gβγ can be recruited and the inactive heterotrimeric complex is formed again (Figure 2) [69]. According to this traditional model of G protein signaling, there is a physical dissociation of Gα and Gβγ upon activation. Some studies point at a rearrangement rather than a dissociation of Gα and Gβγ subunits upon receptor activation. For example, Bünemann et al. found an increase in FRET signal upon receptor activation in living cells instead of the expected decrease if Gα and Gβγ subunits would dissociate [70].

G proteins are usually grouped into four main classes based on similarities between the Gα units: G_s_, G_i/o_, G_q/11_ and G_12/13_ [71]. These classes distinctively affect specific second messenger pathways. A_1_Rs couple to several members of the pertussis toxin (PTX) sensitive G_i/o_ group, namely G_i1_, G_i2_, G_i3_ and G_o1_ [72,73]. G_i_ proteins are named in accordance with their inhibitory effect on adenyl cyclase. The G_o_ protein was discovered later, during the purification of G_i_ proteins, and was named as the ‘other’ GTP-binding protein. It is now known that G_o_ is 5 to 10 times more abundant than other G_i_ proteins and is the most abundant G protein in the CNS [74]. It is worth noting that A_1_Rs can also interact with other G proteins. In Chinese hamster ovary (CHO) cells, the A_1_R can also activate G_S_ and G_q_ proteins depending on the agonist used [75]. It has been suggested that agonist-specific conformations of the receptor lead to differential activation of G_i/o_, G_s_ or G_q_. Yet, coupling to G_i/o_ proteins remains the most prominent way of A_1_R signaling and the neuronal inhibitory effects of A_1_R activation are thus traditionally viewed to be mainly mediated by this group of G proteins.

### 5.2. Adenyl Cyclase and Phospholipase C

When A_1_Rs are being discussed in literature, their effects are mostly commonly attributed to downstream activation of two major signaling pathways: the adenyl cyclase (AC) pathway and the phospholipase C (PLC) pathway [11,22,76]. The former is the most prominent and well-known A_1_R–dependent signaling pathway: A_1_R activation leads to inhibition of AC and as a result less ATP is converted to the second messenger cyclic AMP (cAMP). Cyclic AMP activates protein kinase A (PKA) which then phosphorylates numerous proteins, including several transcription factors such as the well-studied cAMP response binding protein (CREB) [77]. The inhibition of AC by G_i/o_-coupled receptors is mediated via Gα_i_ subunits, but not by the α- subunit of the more abundant G_o_ proteins [74]: purified GTP-bound Gα_i1_, Gα_i2_ and Gα_i3_ units can suppress AC activity, while Gα_o_ cannot [78]. This was also confirmed in a later experiment with mutationally activated Gα units, where Gα_i1_, Gα_i2_, Gα_i3_ but not Gα_o_ inhibited cAMP accumulation [79].

Regulation of the PLC pathway by A_1_Rs is less straightforward as there are both reports of A_1_R-dependent stimulation and inhibition of PLC activity. Phospholipase C enzymatically cleaves the membrane phospholipid phosphatidylinositol 4,5-bisphosphate (PIP_2_) into diacylglycerol (DAG) and inositol 1,4,5-trisphosphate (IP_3_), which both function as important second messengers [80,81]. Diacylglycerol activates protein kinase C (PKC), which phosphorylates a variety of intracellular proteins, while IP_3_ binds to IP_3_-gated calcium channels on the membrane of the endoplasmic reticulum. PIP_2_ regulates the activity of several membrane-bound ion channels, mostly increasing their activity [82]. By hydrolyzing PIP_2_, PLC thus regulates those channels in the opposite way.

Most often, it is stated that A_1_Rs activate PLC-dependent signaling [11,22,76]. Studies in a smooth muscle cell line (DDT_1_ MF2) showed increased formation of IP_3_ and DAG upon administration of a selective A_1_R agonist, increasing PKC activity and mobilizing intracellularly stored Ca^2+^ [83,84]. This positive modulation was confirmed in CHO cells transfected with the rat or human A_1_R gene [85,86]. These cells showed an increase in IP_3_ formation and Ca^2+^ mobilization in response to A_1_R agonists. When A_1_R-expressing cells are transfected with a scavenger of Gβγ the A_1_R induced increase in IP_3_ formation is abolished [87]. This is in line with earlier studies showing that purified Gβγ units directly regulate the activity of PLC [88,89]. However, studies investigating the inhibitory effects of adenosine with CNS-derived tissue found conflicting results. In accordance with the enhancing effect on PLC activity seen in non-neuronal preparations, A_1_R stimulation in guinea-pig cerebral cortical slices [90] and rat striatal slices [91] further augmented histamine-induced IP_3_ accumulation. Yet, in mouse cerebral cortical slices [92] stimulation of A_1_Rs resulted in decreased histamine-induced IP_3_ formation. Additionally, in rat hippocampal slices selective A_1_R agonists inhibit PLC basal activity through G_i/o_ proteins [93,94]. As will be discussed in the next sections, either an increase or decrease in PLC activity could mediate the inhibitory effects of A_1_R activation.

### 5.3. Main Inhibitory Effects

The inhibitory mechanisms through which A_1_Rs exert their anticonvulsive effect occur in two major ways: by decreasing the excitability of neurons via hyperpolarization and through suppressing neurotransmission.

#### 5.3.1. Hyperpolarization

Hyperpolarization of neurons is considered one of the most important mechanisms contributing to A_1_R-mediated seizure suppression. Stimulation of A_1_Rs opens postsynaptic K^+^ channels resulting in potassium efflux. This increased K^+^ conductance decreases the membrane potential and antagonizes membrane depolarization, rendering neurons less excitable. This adenosine-induced hyperpolarization was already demonstrated in 1982 through intracellular recordings in rat hippocampal slices. The investigators already suggested an increase in K^+^ conductance as mechanism [95]. This suggestion was confirmed by several studies a few years later: under voltage clamp, adenosine elicited outward K^+^ currents in mouse striatal neurons and in rat CA1 hippocampal neurons [96,97]. The observed hyperpolarization, between 2 and 10 mV in amplitude, was relatively slow as the outward K^+^ current only peaked 1–2 s after adenosine application [96].

**Figure 3:**
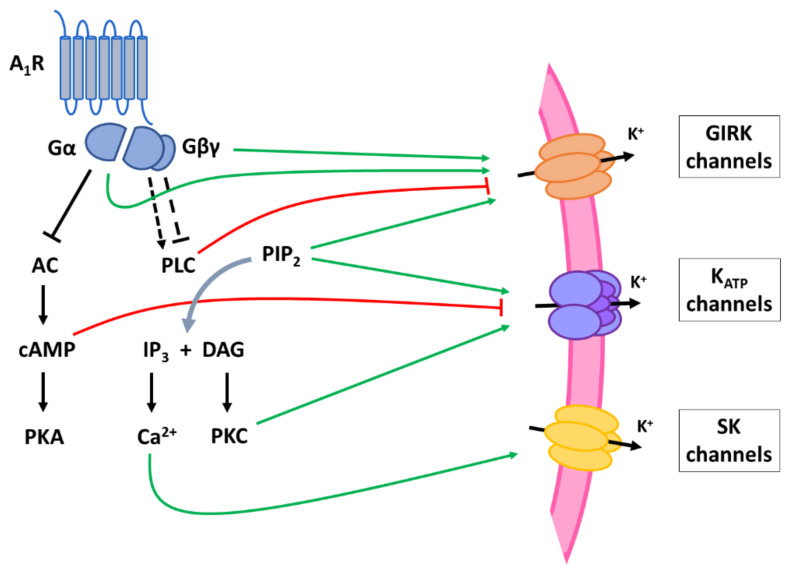
Schematic representation of the signaling pathways involved in increased K^+^ permeability and hyperpolarizing effects of A_1_R activation. A_1_Rs activate G protein-coupled inwardly rectifying K^+^ (GIRK) channels directly via the G protein subunits (Gα and Gβγ) or indirectly by inhibiting PLC activity. A_1_Rs increase ATP-sensitive K^+^ (K_ATP_) channel activity by inhibiting the AC/cAMP pathway or by both stimulating (via increased PKC) or inhibiting (via increased PIP_2_) the PLC pathway. A_1_R-induced IP_3_ stimulation activates small conductance Ca^2+^-activated K^+^ (SK) channels by increasing intracellular Ca^2+^ concentration. The pathway underlying activation of two-pore domain K^+^ (K2P) channels is unknown and therefore not presented here. AC: adenyl cyclase; cAMP: cyclic adenosine monophosphate; PKA: protein kinase A; PLC: phospholipase C; PIP_2_: phosphatidylinositol 4,5-bisphosphate; IP_3_: inositol 1,4,5-trisphosphate; DAG: diacylglycerol; PKC: phosphokinase C.

Explained in more in detail in the following sections, follow-up studies identified the specific channels responsible for this A_1_R-mediated outward K^+^ current such as G protein-coupled inwardly rectifying K^+^ (GIRK) channels and ATP-sensitive K^+^ (K_ATP_) channels (Figure 3). Moreover, small conductance Ca^2+^-activated K^+^ channels and two-pore domain K^+^ channels were reported to be activated by A_1_R signaling, respectively, in retinal ganglion cells [98] and mitral cells [99].

##### GIRK Channels

Soon after discovering the adenosine-activated K^+^ current in striatal neurons, Trussel and colleagues reported this current to be dependent on PTX-sensitive G_i/o_ proteins and GTP [100]. This indicated that adenosine can also activate GIRK channels in neurons, as was already demonstrated for heart muscle cells. In rat CA3 hippocampal neurons adenosine-induced activation of GIRK currents could be blocked with A_1_R antagonists, while A_1_R agonists mimicked the hyperpolarizing effect of adenosine, indicating that A_1_Rs are mediating adenosine-induced activation of GIRK channels [101]. GIRK channels (also known as Kir3 channels) belong to a large family of inwardly rectifying K^+^ channels. The term ‘inward rectification’ refers to the property of these channels to conduct larger inward currents at membrane voltages negative to the K^+^ equilibrium potential (E_K_) than outward currents at voltages positive to this potential. Since the membrane potential of neurons under physiological conditions is positive to E_K_, the opening of Kir channels results in a small outward K^+^ current [102]. Four different GIRK subunits (GIRK1-4, or Kir3.1-4) are expressed in mammals and assemble into homo- or heterotetramers to form functional GIRK channels. In the brain, the subunits GIRK1-3 are the most common [103]. A wealth of research indicates that direct binding of Gβγ is mainly responsible for opening of GIRK channels upon G-protein activation [102]. For instance, in *Xenopus* oocytes only co-expression of GIRK with Gβγ units, but not with Gα, resulted in sustained GIRK channel activity [104]. As well, binding sites for Gβγ could be identified in the N- and C- terminal domains of GIRK1 subunits that are important in GIRK channel activation [105]. However, the Gα subunit plays an important regulatory role. Binding of Gα_i/o_ affects receptor specificity so that only Gβγ dimers derived from G_i/o_ proteins can activate GIRK channels [106]. It also controls gating of GIRK channels, with Gα keeping the basal channel activity low [107,108]. Hence, A_1_R stimulation leads to the activation of GIRK channels in neurons via direct binding of both G protein subunits. Additionally, the PLC pathway is involved in regulation of GIRK channel activity. PLC reduces GIRK channel activity by depleting PIP_2_, which acts as a positive modulator of GIRK channels [103], and by inducing PKC-mediated phosphorylation of GIRK channels [109,110]. A_1_R activation in neurons can reduce PLC activity (see higher) and its inhibitory effects on GIRK channel function, resulting in a net inhibition of neurons.

##### K_ATP_ Channels

Similarly to the GIRK channels, signaling of A_1_Rs to K_ATP_ channels was first discovered in myocytes, soon followed by a study demonstrating that these channels also open in response to adenosine in CA1 hippocampal neurons of the rat [111]. Glibenclamide, a K_ATP_ channel blocker, suppressed the adenosine-induced hyperpolarization of these neurons. A later study in neurons of the substantia nigra delivered further proof that these channels are activated by the A_1_R: selective A_1_R agonists induced an outward K^+^ current sensitive to tolbutamide, another K_ATP_ blocker, while a selective antagonist abolished these effects [112]. K_ATP_ channels also belong to the Kir superfamily and conduct an inwardly rectifying K^+^ current that is inhibited by intracellular ATP. These hetero-octamer channels are composed of four Kir6 subunits (Kir6.1 or Kir6.2) and four sulfonylurea receptor (SUR) subunits (SUR1 or SUR2), with the Kir6 subunits forming the pore while the SUR subunits serve a regulatory role. Binding of ATP to the cytoplasmic domain of Kir6 subunits closes the channel [113]. When ATP concentrations drop, the channels open and hyperpolarize the cell membrane. This way, K_ATP_ channels generally respond to the metabolic activity of cells. However, the sensitivity to the ATP blockade can also be modulated by other proteins, allowing K_ATP_ channels to open in response to external signals regardless of major changes in ATP concentration. To date, the exact mechanism by which A_1_Rs modulate ATP gating of these K^+^ channels remains unknown. Studies in cardiac myocytes point at a role for G_i/o_ proteins as K_ATP_ channels could be activated by application of GTP-bound Gα_i1-3_ units when the channels were closed by intracellular ATP [78,114,115]. In one of these studies, the Gα_o_ subunit was reported to have no effect [114], though in the other studies Gα_o_ was just as effective as the Gα_i_ units [78,115]. At high concentration the Gβγ subunit could also activate K_ATP_ channels, resulting in more potent effects compared to activation by equimolar levels of Gα units [78]. In cardiac myocytes, adenosine activates K_ATP_ channels through PLC-induced activation of PKC, which phosphorylates the Kir6.2 subunit resulting in increased opening probability of the K^+^ channel [116,117]. In neurons it has yet to be demonstrated that this signaling pathway plays a role in the A_1_R-mediated activation of K_ATP_ channels but it is likely that G-protein dependent second messenger pathways are also mediating K_ATP_ channel opening upon A_1_R activation. An increase in PLC/PKC activity is thus likely to be involved in the modulation of neuronal K_ATP_ channels. However, similar to GIRK channels, also inhibition of PLC activity by A_1_Rs (reported by some studies in neurons, cfr. Section 5.2) can potentiate K_ATP_ channel function since PIP_2_ increases the open probability of these channels [113]. Moreover, the AC/cAMP pathway could modulate neuronal K_ATP_ channels. One study has reported a cAMP-dependent modulation of K_ATP_ channels by adenosine in breathing neurons of the pre-Bötzinger complex [118]. The activity of these neurons displays a spontaneous respiratory rhythm, which is decreased by A_1_R stimulation and an accompanying increase in K_ATP_ channel activity. The effects of A_1_Rs on K_ATP_ channel and respiratory rhythm were neutralized by elevation of the intracellular cAMP concentration. These results suggest that inhibition of cAMP formation by A_1_Rs is involved in the activation of K_ATP_ channels, but so far this has not been studied in any other neuronal cells.

##### Small Conductance Ca^2+^-Activated K^+^ Channels

In retinal ganglion cells, small conductance Ca^2+^-activated K^+^ (SK) channels are mediating adenosine-evoked hyperpolarization next to GIRK channels [98]. Indeed, both a GIRK channel blocker (rTertiapin-Q) and a SK channel blocker (apamin) partially inhibited the outward current seen in whole-cell patch-clamp recordings. As their name implies, small conductance Ca^2+^-activated K^+^ channels are activated by an increase in intracellular calcium. Their high Ca^2+^ sensitivity is conferred by calmodulin, bound to the intracellular C terminus of the SK channel. Binding of Ca^2+^ to calmodulin induces opening of the channel, resulting in an outward K^+^ current [119]. The SK component of the A_1_R-induced current in retinal ganglion cells was blocked by IP_3_ receptor antagonists [98]. This would suggest that PLC-mediated formation of IP_3_ induces the release of Ca^2+^ from intracellular stores, which activates the SK channels.

##### Two-Pore Domain K^+^ Channels

A recent study reported that the adenosine-mediated hyperpolarization of mitral cells (projection neurons of the olfactory bulb) was partially blocked by two-pore domain K^+^ (K2P) channel inhibitors (bupivacaine and halothane) [99]. This is a large family of background K^+^ channels which stabilize the negative resting membrane potential. A functional K2P channel consists of two subunits, each of which contains two pore domains (hence the name). The activity of these channels is regulated by a wide variety of parameters; some respond to changes in pH or temperature for example [120]. However, some subfamilies of K2P channels are also known to be regulated by GPCRs. For instance, stimulation of the PLC pathway by Gq-coupled receptors inhibits TASK (TWIK-related acid-sensitive K^+^ channel) and TREK (TWIK-related K^+^ channel) subfamilies, but activates channels of the TRESK (TWIK-related spinal cord K^+^ channel) subfamily. TREK channels are also inhibited by an increase in cAMP, which is counteracted in case of Gi signaling [121]. However, the specific subtype of the K2P channels that were activated by A1Rs in the study on mitral cells could not be identified and thus the pathway responsible for their activation is not known.

#### 5.3.2. Suppression of Synaptic Transmission

**Figure 4:**
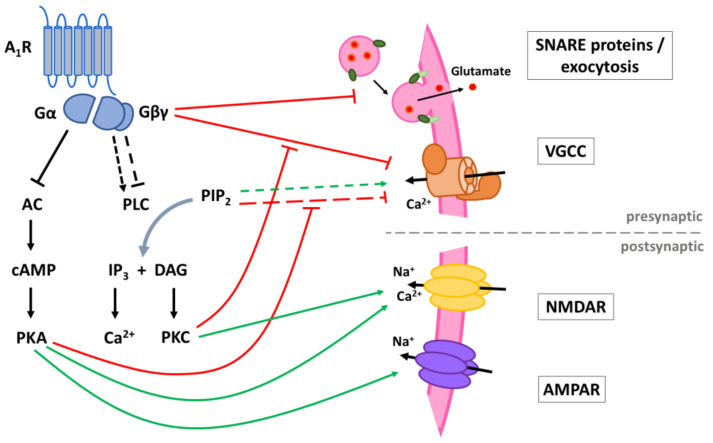
Schematic representation of the signaling pathways involved in the suppression of neurotransmission by adenosine A_1_ receptors (A_1_R). A_1_Rs suppress neurotransmitter release in a Ca^2+^-dependent way by inhibiting voltage-gated Ca^2+^ channels (VGCCs) via Gβγ. Additionally, VGCCs are inhibited through reduced PLC signaling resulting in reduced disinhibition by PKC and increased inhibition by PIP_2_. Inhibition of PKA activity by A_1_R also enhances PIP_2_-mediated inhibition of VGCCs. Through binding of Gβγ to SNARE proteins, A_1_Rs also suppress neurotransmitter release in a Ca^2+^-independent way. Postsynaptic NMDA (NMDAR) and AMPA receptor (AMPAR) function is negatively modulated by A_1_Rs through inhibition of PKA and PKC activity. AC: adenyl cyclase; cAMP: cyclic adenosine monophosphate; PKA: protein kinase A; PLC: phospholipase C; PIP_2_: phosphatidylinositol 4,5-bisphosphate; IP_3_: inositol 1,4,5-trisphosphate; DAG: diacylglycerol; PKC: phosphokinase C.

Besides dampening neuronal excitation through opening of potassium channels, presynaptic and postsynaptic A_1_Rs also antagonize excitation by directly modulating the synaptic transmission (Figure 4). Presynaptically, A_1_R stimulation reduces the release of glutamate and other neurotransmitters in a Ca^2+^-dependent and -independent manner. Postsynaptically, A_1_R stimulation interferes with the function of NMDA (NMDARs) and AMPA receptors (AMPARs), both ionotropic glutamate receptors mediating fast excitatory neurotransmission.

The following paragraphs of this section will focus on excitatory neurotransmission. Nevertheless, it should be mentioned that A_1_Rs have also been found to reduce inhibitory GABAergic transmission in several brain areas [122]. Such modulation of GABAergic transmission could act as a complementary mechanism to control excitation of neuronal circuits. For instance, A_1_Rs have been demonstrated to suppress tonic GABAergic inhibition of interneurons in the hippocampus [123]. Disinhibition of these interneurons results in increased inhibition of pyramidal neurons, thus contributing to a decrease in hippocampal network excitability [124].

##### Inhibition Ca^2+^-Dependent Neurotransmitter Release

Besides modulation of K^+^ channels, the effects of A_1_Rs on presynaptic Ca^2+^ channels are probably the best known explanation for the inhibitory/anticonvulsive effects of these receptors. Action potentials reaching the presynaptic terminal trigger opening of voltage-gated Ca^2+^ channels (VGCCs) and the strong transient increase in intracellular Ca^2+^ nearby the VGCC (Ca^2+^ microdomains) triggers exocytosis of synaptic vesicles and neurotransmitter release [125]. Activation of A_1_Rs suppresses this evoked neurotransmission by inhibiting the Ca^2+^ influx via VGCCs.

VGCCs are Ca^2+^ channels that open in response to large (high-voltage activated; HVA channels) or small (low-voltage-activated; LVA channels) depolarizations of the membrane potential. All VGCCs are composed of a pore-forming and voltage-sensitive α1 subunit, consisting of four transmembrane domains. In case of HVA channels, the α1 subunit co-assembles with ancillary α2δ and β subunits. LVA channels, on the other hand, function as monomeric channels [126]. Based on differences in the α1 subunit, VGCCs are divided over three families: Cav1, Cav2 and Cav3. The Cav3 family makes up the group of low voltage-activated T-type Ca^2+^ channels, while Cav1 and Cav2 belong to the group of HVA channels. The Cav1 family exists of four different types of L-type Ca^2+^ channels (Cav1.1-1.4). The Cav2 family contains three members, each corresponding to a different type of VGCC: the P/Q-type (Cav2.1), the N-type (Cav2.2) and the R-type (Cav2.3) channels. The P/Q-type and the N-type channels are primarily responsible for the initiation of fast synaptic transmission and therefore closely interact with proteins of the synaptic vesicle release complex [126]. Specifically, these two VGCC types are inhibited by A_1_Rs.

Initial studies demonstrated reduced depolarization-evoked Ca^2+^ currents in the soma of neurons. The authors of these studies suggested this might be the mechanism for-adenosine-induced inhibition of neurotransmitter release if similar effects are present at synaptic terminals [127,128,129]. Indeed, studies in hippocampal slices confirmed that A_1_R agonists reduce presynaptic voltage-dependent Ca^2+^ currents at hippocampal synapses [130,131]. Pretreatment with ω-conotoxin GVIA (N-type VGCC blocker) attenuated the effect of adenosine on Ca^2+^ currents in superior cervical ganglion neurons and in hippocampal slices [129,131]. Interestingly, in these studies P/Q-type channels did not seem to play an important role since pretreatment of the neuronal preparations with ω-agatoxin IVA (P/Q-type VGCC blocker) did not result in any significant changes. However, later studies revealed that P/Q-type channels are also modulated by A_1_Rs at hippocampal synapses. The relative contribution of P/Q-type channels to an adenosine-induced decrease in Ca^2+^ current was similar to that of N-type channels at mossy fiber synapses [132]. Another study used ω-conotoxin MVIIC (another P/Q-type blocker) together with ω-conotoxin GVIA in hippocampal synaptosomes to demonstrate the role of P/Q-type and N-type channels [133]. When using a combination of both blockers, adenosine could even further decrease the release of glutamate at hippocampal nerve terminals, suggesting other non-identified VGCCs or other mechanisms (as will be discussed below) may also be involved.

Early studies demonstrated that the activation of PTX-sensitive G_i/o_ proteins is essential for the effects of adenosine on VGCCs and neurotransmission. Activation of G-proteins through the application of GTP-γS in chick sensory neurons mimicked adenosine-induced inhibition of Ca^2+^ currents [128], while PTX-based inhibition of G_i_ proteins abolished the inhibitory effects of adenosine on depolarization-induced Ca^2+^ currents in ganglion neurons [129] and glutamate release by cerebellar neurons [134]. Selective expression of Gβγ units mimicked the effects of G_i/o_-coupled GPCRs on P/Q- and N-type channels indicating that Gβγ is directly involved in the inhibition of Cav2 channels [135,136,137]. The α1 units of these VGCCs indeed possess binding sites for Gβγ in the linker between domain I and domain II that, together with the N-terminal region, form an important interaction site with the Gβγ unit [138,139]. Binding of Gβγ to this interaction site stabilizes the closed conformation of the VGCCs. This direct form of inhibition by G proteins is voltage-dependent as strong membrane depolarization causes a brief dissociation of Gβγ from the channel [135].

Slower, voltage-independent regulation of VGCCs through G-protein mediated second messenger pathways are also in play. The PLC pathway increases P/Q- and N-type VGCC function through PKC-mediated phosphorylation of the domain I-II linker which antagonizes Gβγ-mediated inhibition of these VGCCs [138,140]. A_1_R-mediated inhibition of the PLC/DAG/PKC pathway reduces this antagonism and supports reduced VGCC activity. The activity of P/Q- and N-type channels is also modulated in two different ways by PIP_2_ [141]. Firstly, binding of PIP_2_ to a presumable high-affinity site stabilizes channel activity. Thus, depletion of PIP_2_ by PLC stimulation results in the closing of VGCCs. Secondly, by binding to another, low-affinity binding site, PIP_2_ would cause the VGCCs to be more reluctant to open, inhibiting currents evoked by small depolarizations. Interestingly, this inhibition is blocked by phosphorylation by PKA. This could explain the enhancement of P/Q- and N-type channel currents mediated by cAMP/PKA [142]. This demonstrates that the AC/cAMP/PKA pathway also regulates VGCCs to some degree.

At this moment, it is unclear to which degree the PLC/DAG/PKC and AC/cAMP/PKA pathways contribute to A_1_R-mediated inhibition of P/Q- and N-type channels. Two early studies could not demonstrate a role of the cAMP/PKA pathway in A_1_R mediated inhibition of VGCC in chick sensory neurons and mossy fibers [128,132]. Modulation of PKC activity did also not alter the effects of adenosine on Ca^2+^ currents in chick sensory neurons [128]. In entorhinal cortex (EC) slices, adenosine-mediated suppression of glutamatergic transmission is reduced after pretreatment with AC or PKA inhibitors, suggesting a significant contribution of AC and PKA inhibition to adenosine-induced suppression [143].

##### Decrease in Spontaneous Neurotransmitter Release

A_1_R signaling also suppresses neurotransmission in a calcium-independent way. When synaptic vesicles spontaneously fuse with the presynaptic membrane, they release small amounts of neurotransmitter which results in miniature postsynaptic currents (mPSCs). In hippocampal slices and cultured hippocampal neurons, the frequency of mPSCs is reduced by applying A_1_R agonists. This inhibition is not affected by Ca^2+^ blockers, indicating that A_1_Rs inhibit some component involved in vesicle release downstream from Ca^2^ entry [144,145].

Although phosphorylation of proteins of the vesicle release complex by PKA and PKC is known to play a role in Ca^2+^-independent regulation of neurotransmission, modulation of the AC or PLC pathways does not seem to be involved in the inhibition of mPSCs by adenosine [146,147]. For several other G_i/o_-coupled GPCRs a crucial role for the Gβγ subunit in inhibiting mPSCs has been demonstrated [148]. For example, the injection of Gβγ in presynaptic terminals mimicked the inhibition of neurotransmission by serotonin without affecting Ca^2+^ influx and when a Gβγ inhibitor was injected the inhibitory action of serotonin was lost [149]. Through further investigation, it has been established that Gβγ subunits directly interact with and most likely block SNARE complex proteins which regulate fusion between synaptic vesicles and the synaptic membrane, thus inhibiting exocytosis [148].

##### Inhibition NMDAR Currents

In hippocampal pyramidal cells [150] and basolateral amygdala neurons [151], whole-cell patch-clamp recordings have shown that NMDAR-mediated currents are inhibited by application of A_1_R agonists. NMDA (*N-methyl-D-aspartate*) receptors are ionotropic glutamate receptors which exist as tetrameric channels composed of two GluN1 subunits along with two GluN2 or GluN3 subunits [152]. Receptor activation does not only require binding of glutamate, but also glycine binding and membrane depolarization for relief of the Mg^2+^ block. Opening of the channel allows influx of cations, including Ca^2+^ due to a high Ca^2+^ permeability. Ca^2+^ entry through NMDARs can initiate signaling cascades that lead to modulation of synaptic strength. This way, NMDARs not only mediate synaptic transmission but also synaptic plasticity [152]. In case of neuronal hyperactivity, however, excessive NMDAR stimulation can lead to maladaptive synaptic changes or to cell death due to extreme Ca^2+^ influx. By their inhibitory effect on NMDARs, A_1_R activation can prevent those deleterious Ca^2+^-mediated effects on top of reducing depolarizing currents.

NMDAR function is regulated by many postsynaptic GPCRs in a complex manner. For several of those GPCRs, such as dopamine receptors or GABA receptors, the molecular mechanisms behind the modulation have been well studied, showing that the signaling pathways involved are very variable between the different GPCRs [153]. Even receptors that couple to the same class of G proteins can have different effects on NMDAR activity; Gα_i_ coupled receptors for example may potentiate or depress NMDAR function [154]. Regarding adenosine receptors, more is known about regulation via A_2A_Rs, which have been reported to be able to both potentiate and inhibit NMDARs dependent on the cell type [153]. Though there is currently no direct evidence on the signaling pathways through which A_1_Rs inhibit NMDAR function, literature provides some indications. Firstly, PKA-induced phosphorylation of the C-terminal domains of GluN1 and GluN2 subunits increases NMDAR currents while blocking PKA activity decreases gating and Ca^2+^ permeability of NMDARs [155,156]. A_1_R-mediated inhibition of the AC/cAMP/PKA cascade could thus be responsible for the decrease in NMDAR currents. Secondly, PKC activity increases opening of NMDARs, reduces the Mg^2+^ block and increases channel expression at the cell surface via upregulation of SNARE-dependent exocytosis [157,158]. Therefore, in cells where A_1_R activation inhibits the PLC/DAG/PKC cascade, it is also likely to result in decreased NMDAR activity.

##### AMPAR Modulation

Modulation of AMPA (*α-amino-3-hydroxy-5-methyl-4-isoxazolepropionic acid*) receptors by the A_1_R has received less attention compared to NMDARs, even though AMPARs predominantly mediate fast excitatory transmission and antagonizing them results in more potent seizure suppression [159]. AMPARs are ionotropic glutamate receptors formed as tetramers from GluA1, GluA2, GluA3 or GluA4 subunits. Unlike NMDARs, most AMPARs are only permeable to Na^+^ and K^+^ ions and not to Ca^2+^. They only require binding of glutamate to open and cause depolarization of the membrane potential. While NMDARs possess a relatively stable expression at synapses, AMPARs are more dynamically expressed and can move into or out of the postsynaptic membrane. This variability in AMPAR expression levels is an important factor in the regulation of synaptic plasticity and is mediated by the Ca^2+^ influx caused by NMDARs [159].

A_1_Rs also modulate AMPAR trafficking independent from their effect on NMDARs. The phosphorylation of certain serine and threonine residues at the C-terminus of AMPAR subunits by several kinases, including PKA and PKC, plays an important role in AMPAR function and trafficking [160]. Especially PKA-mediated phosphorylation of Ser845 in GluA1 is key in AMPAR regulation. A_1_Rs maintain an inhibitory tone on Ser845 phosphorylation by inhibiting AC in several regions of the rat brain. Inhibition of A_1_R signaling under basal adenosine concentrations increases Ser845 phosphorylation and potentiates AMPAR currents while selective A_1_R activation reduces AMPAR currents in hippocampal slices [161,162]. Furthermore, it was also reported that A_1_Rs decrease the agonist affinity of AMPARs [162].

In addition, A_1_Rs can also reduce AMPAR expression through protein phosphatases (PP) which dephosphorylate the serine residues involved in receptor trafficking. A study in rat hippocampal slices demonstrated that GluA1 and GluA2 internalization after prolonged A_1_R stimulation is mediated by PP1, PP2A and PP2B using selective phosphatase inhibitors [163]. The signaling pathway for activation of phosphatases by A_1_Rs possibly involves mitogen-activated protein kinases (MAPKs) since PP2A is activated by p38 MAPK upon A_1_R stimulation [164,165] and inhibition of p38 MAPK and JNK (c-Jun N-terminal kinase) prevents GluA2 subunit internalization [166]. This signaling pathway will be discussed in more detail in the following section.

### 5.4. Other Signaling Pathways and Their Effects

The effects discussed above are the most well-known and major mechanisms by which the A_1_R leads to neuronal inhibition and anticonvulsant effects. They immediately affect neuronal excitability and are all mediated by the AC or PLC pathway and/or directly by G protein subunits. However, activation of A_1_Rs can result in several additional effects through activation of a variety of other signaling pathways. Below, we will outline a couple of other important pathways affected by the A_1_R, together with their relevance in the context of epilepsy.

#### 5.4.1. Activation of MAP Kinases

MAP kinases are a protein family consisting of three main groups: the extracellular signal-regulated kinases (ERKs), the c-Jun N-terminal kinases (JNKs) and the p38 MAPKs/stress-activated protein kinases (SAPKs) [167]. These kinases are well known for their role in cell proliferation, cell growth and cell death, but they are involved in many more cellular functions. MAPKs are activated by various extracellular stimuli through a cascade of protein kinases; the MAPK kinases (MAPKK) and the MAPKK kinases (MAPKKK). The canonical pathway for activating MAPKs involves binding of mitogens (hence the name) or growth factors to receptor tyrosine kinases, followed by receptor dimerization and cross-autophosphorylation. This receptor phosphorylation triggers a signaling cascade via various intermediate proteins upstream from the MAPKKK.

**Figure 5:**
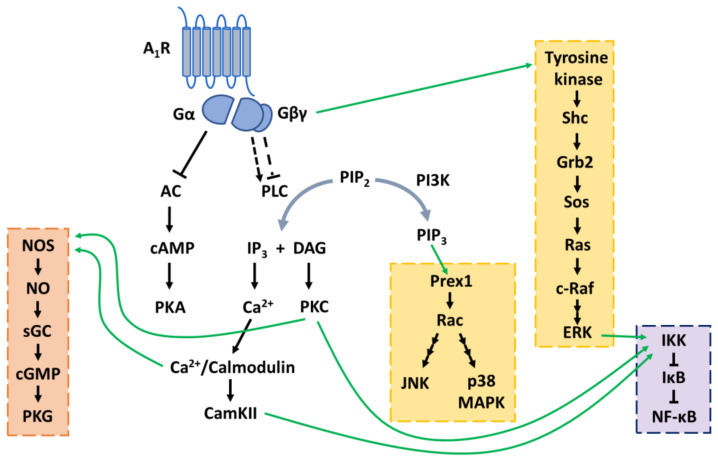
Activation of MAPK pathways (highlighted in yellow), the guanyl cyclase pathway (highlighted in orange) and nuclear factor-κB (highlighted in purple) by the adenosine A_1_ receptor (A_1_R). AC: adenyl cyclase; cAMP: cyclic adenosine monophosphate; PKA: protein kinase A; PLC: phospholipase C; PIP_2_: phosphatidylinositol 4,5-bisphosphate; IP_3_: inositol 1,4,5-trisphosphate; DAG: diacylglycerol; PKC: phosphokinase C; CamKII: calmodulin-dependent protein kinase II; PI3K: phosphatidylinositol 3-kinase; PIP_3_: phosphatidylinositol 3,4,5-trisphosphate; JNK: c-Jun N-terminal kinase; ERK: extracellular signal-regulated kinase; p38 MAPK: p38 mitogen-activated protein kinase; NOS: nitric oxide synthase; NO: nitric oxide; sGC: soluble guanylyl cyclase; cGMP: cyclic guanosine monophosphate; PKG: protein kinase G; IKK: IκB kinase; NF-kB: nuclear factor-κB.

GPCRs, including all adenosine receptors, can also activate MAPKs by tapping into this pathway [168]. All three groups of MAPKs are activated by A_1_Rs (Figure 5) [169,170], of which A_1_R-mediated activation of ERKs is best studied. It was first discovered in immortalized kidney fibroblasts (COS-7 cells) that ERK1 is activated by A_1_Rs (and other G_i_-coupled receptors) via Gβγ subunits [171]. Further studies in CHO cells showed that Gβγ activates tyrosine kinase which then phosphorylates Shc. Phosphorylated Shc forms a complex with Grb2 followed by consecutive activation of Sos, Ras and c-Raf. c-Raf is the MAPKKK that leads to ERK1/2 activation [172]. However, this pathway cannot be generalized to all cell types since a study in a smooth muscle cell line showed that tyrosine kinase inhibition did not block A_1_Rs-mediated activation of ERK1/2, but phosphatidylinositol 3-kinase (PI3K) inhibitors did. [169]. PI3K could theoretically mediate JNK and p38 MAPK activation as well. Phosphatidylinositol 3,4,5-trisphosphate (PIP_3_), formed by PI3K, activates the guanine–nucleotide exchange factor Prex1 which in turn activates Rac [173]. Rac is a GTPase is involved in signaling cascades leading to JNK and p38 MAPK activation [174].

Activation of MAPK pathways by A_1_Rs most likely contributes to the seizure-suppressive effects of adenosine. JNK and p38 MAPK are involved in A_1_R-mediated suppression of synaptic transmission in the CA1 region of the hippocampus by mediating for example AMPAR internalization via activation of phosphatases (see AMPAR Modulation, Section 5.3.2) [166,175]. Furthermore, MAPKs are involved in protective mechanisms against seizure-induced cell death. Acute seizures in rats induce ERK and p38 MAPK activation in the hippocampus. Blocking these MAPKs aggravates neuronal degradation caused by a subsequent status epilepticus [176]. However, excessive activation of MAPK pathways can also cause negative effects. For instance, constitutive ERK activation increases NMDAR activity by augmenting GluN2 subunit protein levels. This increases neuronal excitability and results in epileptic seizures [177]. Additionally, MAPKs are implicated in epileptogenesis by affecting RNA-binding proteins. In this way, the overactivation of MAPKs can lead to aberrant expression of synaptic proteins [178]. Acute activation of MAPKs by the A_1_R could thus be beneficial, but overactivation becomes more detrimental.

#### 5.4.2. Guanyl Cyclase Pathway

Another pathway activated by the A_1_R is the soluble guanylyl cyclase (sGC) or the nitric oxide (NO)/cyclic guanosine monophosphate (cGMP) pathway. Nitric oxide release upon activation of nitric oxide synthase (NOS) activates sGC, which converts GTP to cGMP. The main effector of cGMP is protein kinase G (PKG). In the CNS, the NO/cGMP pathway exerts many functions including modulation of neuronal excitability and synaptic transmission [179,180].

By blocking NOS, sGC and PKG, Cascalheira et al. demonstrated that A_1_R-induced inhibition of neurotransmission in the CA1 region of hippocampal slices is partly mediated by the NO/cGMP pathway [181,182]. In cardiomyocytes, the activation of A_1_Rs stimulates NOS through activation of PLC and subsequent increase in Ca^2+^/calmodulin and PKC activity [183]. Moreover, the A_1_R-induced phosphorylation of p38 MAPK is prevented by inhibitors of the cGMP pathway, providing a link between A_1_R—induced activation of NO/cGMP and MAPKs [165]. It is yet to be determined whether these mechanisms apply to neurons as well.

#### 5.4.3. Modulation of Nuclear Factor-κB and Brain-Derived Neurotrophic Factor

Stimulation of A_1_Rs can also produce more delayed effects by influencing gene expression. As an example, we will briefly discuss the effects of the A_1_R on the transcription factor nuclear factor-κB (NF-κB) and one of its target gene products—brain-derived neurotrophic factor (BDNF)—since these factors are involved in epilepsy.

NF-κB is an inducible transcription factor that regulates the expression of hundreds of genes involved in inflammation, immunity, cell survival and cell differentiation. NF-κB is present in the cytoplasm in an inactive state as long as it is associated with its inhibitor; IκB (inhibitor of κB). Phosphorylation of IκB triggers its ubiquitination and degradation and activation of NF-κB [184]. NF-κB activation can be initiated by a large number of environmental stimuli, such as bacterial products or UV light, as well as by a variety of GPCRs, including the A_1_R. It was shown, for example, that application of adenosine in rat basal forebrain slices increased the amount of NF-κB bound to DNA. This was significantly reduced by pretreatment of the slices with an A_1_R antagonist [185]. On a side note, NF-κB can also bind to the promoter sequence of the A_1_R gene and increase A_1_R expression [186]. The signaling pathway responsible for the activation of NF-κB by A_1_Rs is not yet clarified. In human lymphoblastoma and embryonic kidney cells [187], A_1_R-induced NF-κB activation was not mediated by G_i/o_ proteins but instead relied on G_16_, a G protein specific to hematopoietic cells [188]. Two cascades were found to be initiated by Gα_16_ and Gβγ: (1) activation of PLC, resulting in activation of PKC and calmodulin-dependent protein kinase II (CaMKII) due to increased Ca^2+^ concentration and (2) activation of the tyrosine kinase c-Src, which initiates a MAPK cascade leading to phosphorylation of ERK via Ras and c-Raf. All three kinases -PKC, CaMKII and ERK-can activate the IκB kinase (IKK) complex which phosphorylates IκB and releases NF-κB. Whether any of these pathways are involved in NF-κB activation in neurons remains to be studied.

In the CNS, NF-κB can have a neuroprotective role, but it is also involved in neurodegeneration. It is believed that a certain level of NF-κB is required to maintain normal neuronal functioning while too low or too high NF-κB levels are pathological [189]. Based on preclinical seizure models, it is not clear whether the activation of NF-κB by A_1_Rs could be beneficial or detrimental. In one rat study, inhibition of NF-κB increased susceptibility for kainic acid induced seizures [190]. However, another study in the same rat model showed a decrease in seizure susceptibility and also found that NF-κB inhibition resulted in decreased expression of P-glycoprotein [191]. NF-κB activation could thus lead to an elevated risk for seizures and increased P-glycoprotein expression, an important multidrug transporter implicated in drug-resistance in epilepsy. These conflicting results demonstrate the complexity of NF-κB signaling, owing to the many possible genes that can be induced by this transcription factor.

One of the genes which expression is affected by NF-κB is BDNF [192]. Induction of NF-κB activity in response to kainic acid administration increases the expression of BDNF both in vitro as in vivo [190,193]. A_1_R stimulation could thus result in upregulation of BDNF via NF-κB. This is supported by a recent study with A_1_R-knock out mice, where BDNF levels after seizure induction were lower in the knock out compared to wild type animals [194]. As neurotrophin, BDNF is important for the growth and survival of neurons during development. In the mature brain, the function of BDNF is less clear. BDNF has been reported to induce phosphorylation of GluN1 subunits of NMDARs, thereby increasing their activity [195]. In the context of epilepsy, indeed, most evidence indicates that BDNF increases neuronal excitability and contributes to epileptogenesis [196,197]. However, there is some evidence that BDNF can have a neuroprotective effect by increasing the expression of the inhibitory neuropeptide Y (NPY) [196,197]. Via its G_i/o_-coupled receptors, NPY also inhibits several types of VGCCs and activates GIRK channels [198,199].

## 6. Conclusions

**Figure 6:**
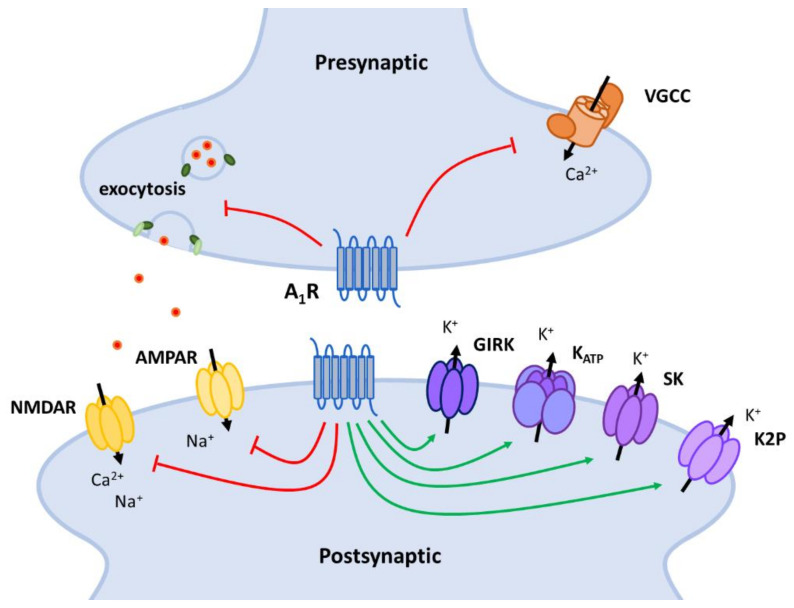
Overview of the pre- and postsynaptic targets of the adenosine A_1_ receptor (A_1_R) through which it mediates its main inhibitory neuromodulatory effects; hyperpolarization via activation of K^+^ channels and suppression of synaptic transmission via inhibition of voltage-gated Ca^2+^ channels (VGCCs) and proteins involved in exocytosis. AMPAR: AMPA receptor; NMDAR: NMDA receptor; GIRK: G protein-coupled inwardly rectifying K^+^ channel; K_ATP_: ATP-sensitive K^+^ channel; SK: small conductance Ca^2+^-activated K^+^ channel; K2P: two-pore domain K^+^ channel.

The importance of the A_1_R, through which the adenosinergic system exerts many of its anticonvulsive and neuroprotective effects, is well established. This review provided an overview of signaling pathways through which A_1_R activation yields those effects. The two principal inhibitory neuronal mechanisms of the A_1_R are well known; (1) membrane hyperpolarization caused by the activation of K^+^ channels and (2) suppression of synaptic transmission via inhibition of VGCCs and synaptic vesicle release (Figure 6). The second messenger systems and molecular mechanisms responsible for the activation or inhibition of these targets, however, remain to be completely unraveled, though evidence indicates important roles of the AC and the PLC pathways, along with the Gβγ subunit. Additional evidence indicates a role for the NO/cGMP pathway and the MAPKs in mediating the inhibitory actions of the A_1_R. Caution must be taken, however, as a major part of the evidence reviewed regarding A_1_R signaling is derived from studies in non-neuronal cells. Beyond acute anticonvulsive effects, it is important to consider that A_1_R activation can result in additional delayed and long-term neuromodulatory effects. These can have an opposite, detrimental effect and potentially aggravate seizure activity. Thus, when developing future epilepsy therapies based on A_1_R stimulation, the aim should be to evoke the immediate inhibitory effects of the A_1_R while avoiding the negative effects of chronic overstimulation.

## Figures and Tables

**Table 1:** Changes in expression levels of A_1_Rs in chronic epilepsy.

Observations in TLE Patients				
Change	Tissue		Detection Method + Results	Ref.
Increased A_1_R expression in human refractory TLE patients	- Excised epileptic temporal lobe tissue refractory TLE patients (*n* = 5) Excised control temporal lobe tissue brain tumor patients (*n* = 6)		- Autoradiographic labeling of A_1_R with [^3^H]CHA - 48% increase in A_1_R binding density	[44]
Decreased A_1_R expression in human refractory TLE patients	- Excised epileptic temporal lobe tissue refractory TLE patients (*n* = 15) - Post-mortem control temporal lobe tissue from non-epileptic subjects (*n* = 9)		- Autoradiographic labeling of A_1_R with [^3^H]CHA - 70% decrease in A_1_R binding density	[45]
**Observations in chronic epilepsy models**				
**Change**	**Animal model**	**Timeframe**	**Detection method + results**	**Ref.**
Decreased A_1_R expression in CA2/CA3 regions of epileptic rats	- Wistar rats - KA i.p. treatment - Hippocampal kindling	1–2 months after treatment	- Immunohistochemical labeling with A_1_R antibody - Near 100% loss of A_1_R immunoreactivity - Decrease in A_1_R immunoreactivity on stimulated but not on contralateral side of kindled animals	[46]
Decreased A_1_R expression in CA1/CA3 regions of epileptic rats	- S-D rats - KA i.p. treatment	30 days after treatment	- Autoradiographic labeling of A_1_R with [^3^H]CHA - 70% decrease in A_1_R density in CA1, 40% decrease in CA3 - related to neuronal degradation	[47]
Decreased A_1_R expression in hippocampal slices of epileptic rats	- Wistar rats - Amygdala kindling	3–4 weeks after treatment	- Autoradiographic labeling of A_1_R with [^3^H]R-PIA - Immunohistochemical labeling with A_1_R antibody - 43% decrease in A_1_R binding density	[48]
Increased A_1_R expression in epileptic mice	- Balb/C mice - PTZ kindling (i.p.)	1–4 weeks after treatment	- Autoradiographic labeling of A_1_R with [^3^H]CHA - >20% increase in A_1_R binding	[49]
Increased A_1_R expression in medial entorhinal cortex slices of epileptic rats	- S-D rats - Hippocampal kindling	2 months after treatment	- qPCR - Immunohistochemical labeling with A_1_R antibody - 378% increase in A_1_R mRNA - 60% increase in A_1_R immunoreactivity	[50]

## Abbreviations

A_1_R	Adenosine A_1_ receptor
A_2A_R	Adenosine A_2A_ receptor
A_2B_R	Adenosine A_2B_ receptor
A_3_R	Adenosine A_3_ receptor
AC	Adenyl cyclase
ADA	Adenosine deaminase
ADK	Adenosine kinase
AMP	Adenosine monophosphate
AMPAR	Α-amino-3-hydroxy-5-methyl-4-isoxazolepropionic acid receptor
ATP	Adenosine triphosphate
BDNF	Brain-derived neurotrophic factor
CamKII	Calmoduline-dependent protein kinase II
cAMP	Cyclic adenosine monophosphate
cGMP	Cyclic guanosine monophosphate
CHO	Chinese hamster ovary
CNS	Central nervous system
CREB	cAMP response binding protein
DAG	Diacylglycerol
ENT	Equilibrative nucleoside transporter
ERK	Extracellular signal-regulated kinase
FRET	Fluorescence resonance energy transfer
Gα	G protein α subunit
Gβγ	G protein βγ subunit
GDP	Guanosine diphosphate
GIRK	G protein-coupled inwardly rectifying K^+^ channel
GPCR	G protein-coupled receptor
GTP	Guanosine triphosphate
HVA	High-voltage activated
IKK	IκB kinase
IκB	Inhibitor of IκB
IP_3_	1,4,5-triphosphate
JNK	c-Jun N-terminal kinase
K2P	Two-pore domain K^+^ channel
K_ATP_	ATP-sensitive K^+^ channel
LVA	Low-voltage activated
MAPK	Mitogen-activated protein kinase
MAPKK	MAPK kinase
MAPKKK	MAPKK kinase
mPSC	Miniature postsynaptic current
NF-κB	Nuclear factor-κB
NMDAR	N-methyl D-aspartate receptor
NO	Nitric oxide
NOS	Nitric oxide synthase
NPY	Neuropeptide Y
PI3K	Phosphatidylinositol 3-kinase
PIP_2_	Phosphatidylinositol 4,5-biphosphate
PIP_3_	Phosphatidylinositol 3,4,5-triphosphate
PKA	Protein kinase A
PKC	Protein kinase C
PKG	Protein kinase G
PLC	Phospholipase C
PP	Protein phosphatase
PTX	Pertussin toxin
SAPK	Stress-activated protein kinase
sGC	Soluble guanylyl cyclase
SK	Small conductance Ca^2+^-activated K^+^ channel
SUR	Sulfonylurea receptor
TASK	TWIK-related acid sensitive K^+^ channel
TREK	TWIK-related K^+^ channel
TRESK	TWIK-related spinal cord K^+^ channel
VGCC	Voltage-gated Ca^2+^ channel
